# Assessment of Deviations of Implants Installed with Prototyped Surgical Guide and Conventional Guide: In Vitro Study

**DOI:** 10.1055/s-0040-1718791

**Published:** 2022-09-05

**Authors:** João Marcelo Meireles Rodrigues, Pâmela Leticia Santos, Gustavo Mendonça, Ana Paula de Souza Faloni, Livia Sertori Finoti, Rogério Margonar

**Affiliations:** 1 Department of Health Sciences, Postgraduation Program in Implantology, School of Dentistry, University of Araraquara, UNIARA, Araraquara, Sao Paulo, Brazil; 2 Division of Prosthodontics, Department of Biologic and Materials Sciences, School of Dentistry, University of Michigan, Ann Arbor, Michigan, United States; 3 Center for Applied Genomics, Children's Hospital of Philadelphia, Philadelphia, Pennsylvania, United States

**Keywords:** dental implants, surgical guides, guided surgery, computed tomography

## Abstract

**Objective** The study aimed to assess the angular and linear deviations of implants installed in mannequins aided by surgical guides produced with the techniques of dual tomography (DT), model-based tomography (MT), and nonprototyped guide.

**Materials and Methods** Implants were installed in mannequins of a partially edentulous maxilla and divided into three groups: Group C ( *n* = 20), implants installed using the conventional technique with flap opening and conventional guide; Group DT ( *n* = 20), implants installed using guided surgery with the dual tomography technique; and Group MT ( *n* = 20), implants installed using the model-based tomography technique. After implant installation, the mannequin was subjected to a computed tomography (CT) to measure the linear and angular deviations of implant positioning relative to the initial planning on both sides.

**Results** There was a higher mean angular deviation in group C (4.61 ± 1.21, *p* ≤ 0.001) than in groups DT (2.13 ± 0.62) and MT (1.87 ± 0.94), which were statistically similar between each other. Similarly, the linear deviations showed group C with the greatest discrepancy in relation to the other groups in the crown (2.17 ± 0.82, *p* = 0.007), central (2.2 ± 0.77, *p* = 0.004), and apical (2.34 ± 0.8, *p* = 0.001) regions.

**Conclusion** The techniques of DT and MT presented smaller angular and linear deviations than the conventional technique with the nonprototyped guide. There was no difference between the two-guided surgery techniques.

## Introduction

The quantity and quality of bones, considering their morphology and density, were the only predictors of success in the osseointegration of dental implants. 1 However, with the advances in research, the reverse planning became crucial for prosthetic predictability and consequently the success of current implantology. Thus, the development of the surgical guide for transferring the reverse planning has been essential. 2 3

The diagnosis of such parameters, which was performed previously with periapical radiographs, 4 panoramic images, 5 and conventional surgical guide, 6 7 8 and considering the advances in technology for preoperative assessment of dental implant candidates, started to offer the use of computed tomography (CT) and prototyped surgical guide as options. 9 10 11 12 13

The CT is a precise and noninvasive technique that allows studying the skeletal facial anatomy in detail, which is based on images of the medullary and cortical bone, its irregular margins, and the relationship of dental roots with the adjacent structures. 5 This technology allowed the virtual planning of the treatment to increase predictability, aid the precision of implant positioning, and potentially reduce surgical morbidity. 14 15

This virtual planning is transferred to the patient through the production of a surgical guide. 16 17 However, studies showing potential complications due to small deviations have also been published. 18 19 To correct similar failures, the techniques for performing guided surgery have been improved, especially with the development of software that allow manipulating the tomography data and materialize them into prototyped guides rather accurately. 20 21

One of these techniques consists of overlapping the tomographic image of the patient with the tomographic guide on the tomographic image of the guide alone, thus allowing the visualization of bone tissue and teeth. This technique is known as dual tomography (DT). 21 22 23 24 25 However, this procedure requires a laboratory phase to produce the tomographic guide and a posterior CT scan of the patient with the guide in position. 25 26 Therefore, several visits and a laboratory are required, demanding a lot of time from the clinician, the patient, and the prosthetist. Moreover, usually the patient has already performed the tomography without the tomographic guide, thus requiring a new examination, which results in higher cost and radiation dose.

Alternatively, aiming to reduce the limitations aforementioned and the distortion of the final surgical guide, a new technique has been developed, which is the model-based tomography (MT). This procedure requires a CT scan of both the patient and the model that will be cutout virtually to obtain a three-dimensional (3D) model able to reproduce soft tissues such as the gingiva, overlapped on the tomography of the patient's mouth, allowing bone assessment. Thus, this technique can reproduce a real condition of the patient's mouth (soft and hard tissues), which would decrease distortion when producing the guide and save time and cost for both patient and professional. However, there are no studies showing the potential deviations after implant installation with prototyped guide obtained by this technique. Another aspect is the lack of comparison with the conventional implant installation technique.

Hence, this study aimed to assess the angular and linear deviations of implants installed in mannequins aided by surgical guides produced with the techniques of DT and MT, and the conventional implant installation with nonprototyped surgical guide.

## Materials and Methods

### Experimental Design

The present study used 10 dental mannequins of the maxilla (Nacional Ossos, Jaú, SP, Brazil) without elements 14, 15, 16, 24, 25, and 26 of similar bone density on both sides.

**Fig. 1:**
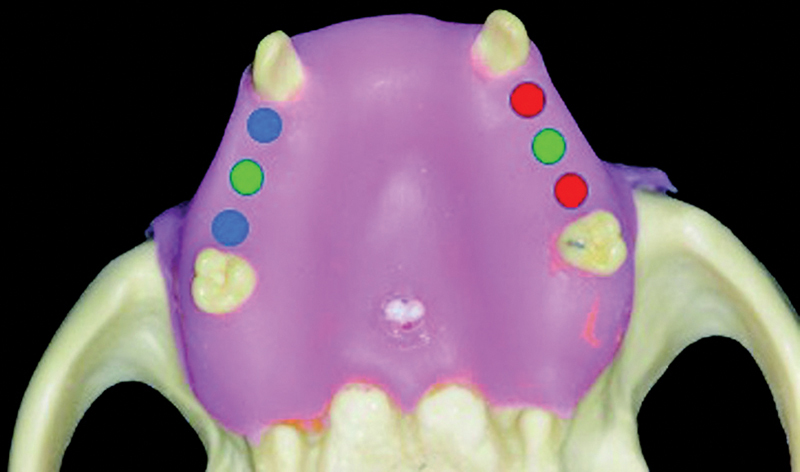
Mannequin used in the study. Group MT: absence of elements 14 and 16, blue; Group C: absence of elements 15 and 25, green; Group DT: absence of elements 24 and 26, red.

The artificial maxillae received three cone morse implants on each side, with 3.5 × 13 mm (UNII Cone Morse - Implacil De Bortoli; São Paulo, SP, Brazil). The groups were divided as follows ( Fig. 1 ):

Group DT ( *n* = 20): Implants installed in the region of teeth 24 and 26 with guided surgery by the DT technique. The surgical procedure was performed with the Raptor surgical kit (Implacil De Bortoli, São Paulo, SP, Brazil).

Group MT ( *n* = 20): Teeth 14 and 16 received implants using the MT technique. The surgical procedure was performed with the Raptor surgical kit (Implacil De Bortoli).

Group C ( *n* = 20): Implants installed conventionally in the region of teeth 15 and 25, using the cylinder set (Implacil De Bortoli) with flap opening and conventional guide.

### Preparation and Virtual Planning

All cone beam computed tomographies (CBCT) were performed in the same iCat Classic scanner (Imaging Sciences International, United States) with 0.25 mm of cutting thickness, 0.25 mm of reconstruction interval, 120 KV, and 36.12 mAs as exposure factors.

#### Group DT: Dual Tomography Technique

**Fig. 2:**
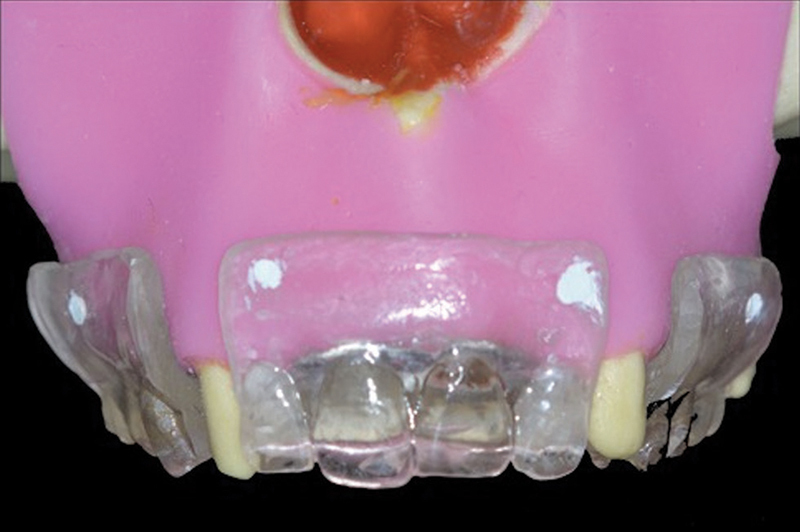
Tomographic guide with radiopaque marks.

A mold with Zetaplus Oral Wash condensation silicone (Zhermack, Italy) was produced for each mannequin, and plaster was poured for obtaining the models and the diagnostic waxing in edentulous spaces. Similarly, a tomographic guide in transparent acrylic resin was produced for each mannequin. Four gutta-percha marks were performed in the buccal groove bottom region ( Fig. 2 ).

After producing this guide, a tomography of the mannequin with the tomographic guide positioned and another one of the guides alone were performed. The images obtained in digital imaging and communications in medicine file (DICOM) were converted into the bioparts extension and overlapped with the Dental Slice Converter software (Bioparts), using the radiopaque points as reference. After joining the images, the virtual planning was performed with the Dental Slice software (Bioparts).

#### Group MT: Model-Based Tomography Technique

**Fig. 3:**
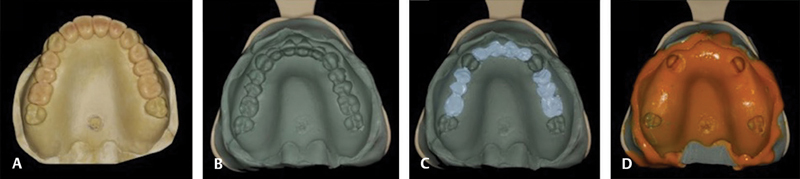
**(A)** Diagnostic waxing in the plaster model of teeth absent from the mannequin; **(B)** dense condensation silicone mold of the plaster model with diagnostic waxing of absent teeth; **(C)** filling of the elements absent from the mannequin with radiopaque material; and **(D)** mold relined in the mannequin with low-density condensation silicone paste.

A mold of the mannequin was produced with Zetaplus Oral Wash condensation silicone (Zhermack, Italy), and plaster was poured for obtaining the models and the diagnostic waxing in edentulous spaces ( Fig. 3A ).

The waxing was molded with Zetaplus Oral Wash condensation silicone (Zhermack, Italy) using a plastic impression tray and only the material with the highest density from the Zetaplus condensation silicone (Zhermack, Italy; Fig. 3B ).

With a molding syringe, the volume of the crowns of teeth to be exposed was filled with R-Silix hyperdense silicone (Bioparts, Brasília, DF, Brazil), which uses the activator of the Zetaplus Oral Wash condensation silicone kit (Zhermack, Italy; Fig. 3C ).

After filling these elements with the hyperdense material, the mold was then relined in the mannequin with the low-density paste from the Oranwash condensation silicone (Zhermack, Italy; Fig. 3D ).

This mold was subjected to tomography and, with the Dental Slice Converter software (Bioparts), a virtual cutout was performed to obtain a computer template and synchronize it with the mannequin image, with the guide positioned for the virtual planning. Having the guide image in the tomography does not interfere with the process, considering that synchronization is performed based on the crowns of the remaining teeth 16, 13, 23, and 27, and the guide was designed on the virtual model. The virtual planning for implant installation was performed in the Dental Slice software (Bioparts).

#### Group C: Flap Opening Technique with Conventional Surgical Guide (Conventional)

The tomographic guide in transparent acrylic resin produced for group DT ( Fig. 2 ) was opened in the regions of teeth 15 and 25, and the buccal flange was removed and transformed into a conventional surgical guide. Considering this is not a prototyped guide, opening the flap to place the implants is required.

### Surgical Guide

All files of the virtual planning were sent to Bioparts (Brasília) for producing the surgical guides by the stereolithography method with the SLA-250/50 printer (3D System, USA), using the Accura 25 resin (3D System ). The guides presented cylindrical perforations in which metal sockets were inserted to transfer the position and inclination of the implants according to the virtual planning.

### Implant Installation

Implants (UNII Cone Morse - Implacil De Bortoli; São Paulo, SP, Brazil) of 3.5 × 13.0 mm were installed using the following sequence of drills: spear, helical 2.0 and 2.8, and lastly the tapered drill of 3.5 × 13.0 mm from the Raptor surgical kit (Implacil De Bortoli, São Paulo, SP, Brazil). In groups DT and MT, the prototyped guides were used for this procedure.

In group C, the conventional guide was used for perforation with the spear and helical 2.0 drills. Next, the guide was removed and the pilot drill was used, namely the helical 2.8 drill from the cylinder set (Implacil De Bortoli).

### Measurements

After installing the implants, the mannequins were again subjected to tomography and the DICOM file was converted into the Dental Slice (Bioparts) for overlapping the images of the virtual planning and final positioning of implants ( Fig. 3 ). The tomographies were performed with the same parameters and in the same device for measuring implant positioning relative to the initial planning on both sides. The following references were captured over the long axis of each implant planned and installed:

- Point in the apical limit of implant D1
- Point is the central region of implant D2
- Point in the crown limit of implant D3
- Direction vector over the long axis of the implant

Then, measurements were taken for the angles formed between the centers of implants planned and installed, and the distances between the top of implants planned and installed (D1), as well as in the center of implants planned and installed (D2) and in the top of implants planned and installed (D3) ( Fig. 3 ).

### Statistical Analysis

The data for angular and linear deviations were subjected to parametric statistical analysis, considering that data adhere to the normality curve, as observed in the Shapiro–Wilk test. The statistical analysis of the data was performed in two steps: one for linear deviations between the implant planned and installed, and another for analyzing angular discrepancies. The multiple comparisons were performed among the three groups using analysis of variance and Tukey's post-test ( *p* < 0.05). All statistical tests were performed with the GraphPad Prism 6.0 software (GraphPad Software; La Jolla, California, United States).

## Results

**Fig. 4:**
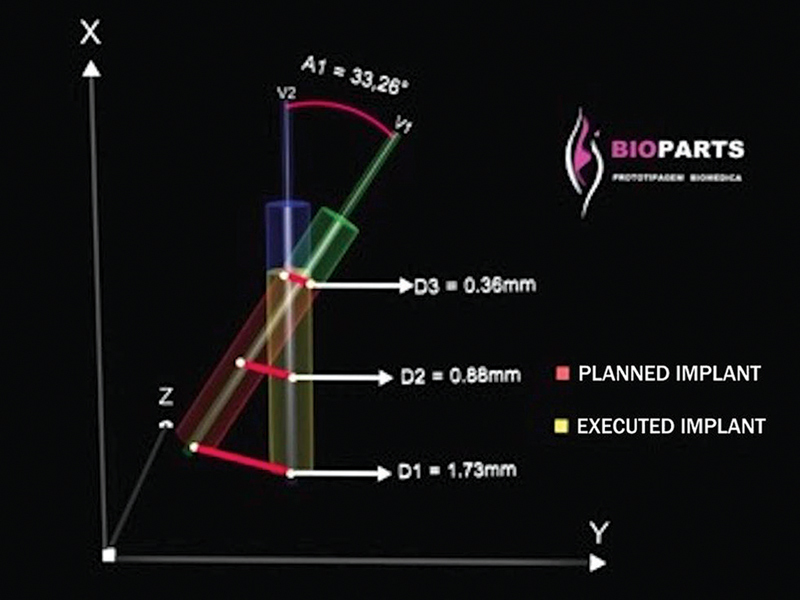
Schematic drawing of the methodology used to assess the discrepancy between pre- and postoperative positions of implants.

The overlapping of pre- and postoperative 3D templates in a virtual environment allowed, analyzing occasional discrepancies between implants planned and installed, according to the methodology previously described. The variations of linear and angular deviations between implants planned and installed may be assessed according to Fig. 4 .

**Fig. 5:**
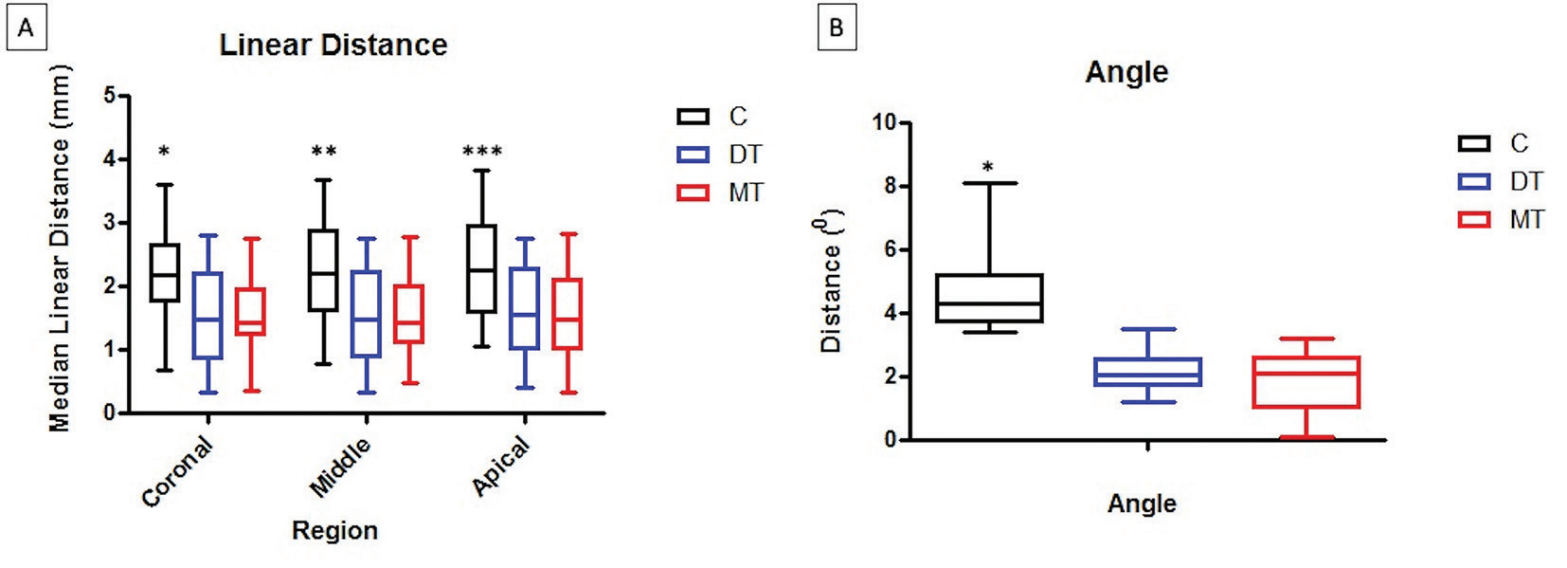
**(A)** Means and standard deviations of the linear distances between implants planned and installed. ANOVA and Tukey ( *p* = 0.05): * *p* = 0.007; ** *p* = 0.001; *p* = 0.004. **(B)** Means and standard deviations of the angular distances between implants planned and installed. ANOVA and Tukey ( *p* = 0.05): * *p* ≤ 0.001. ANOVA, analysis of variance.

According to the tomographic analysis, the implants installed using the flap opening technique with conventional surgical guide presented a linear discrepancy in the crown region (2.17 ± 0.82, *p* = 0.007) compared with groups DT (1.53 ± 0.75) and MT (1.53 ± 0.54), as well as in the central region: groups C (2.2 ± 0.77, *p* = 0.004), DT (1.55 ± 0.76), and MT (1.5 ± 0.6), and in the apical region: groups C (2.34 ± 0.8, *p* = 0.001), DT (1.6 ± 0.76 = 5), and MT (1.5 ± 0.64). This confirms a larger linear deviation between the virtual planning and execution in all points assessed in group C relative to groups DT and MT, considering the last two groups presented similar results between each other in all points assessed for the distance between implants planned and installed ( Fig. 5A ).

For assessing the angular measurements, the results obtained also showed a higher discrepancy in group C (4.61 ± 1.21, *p* ≤ 0.001) between planning and execution in all points assessed when compared with groups DT (2.13 ± 0.62) and MT (1.87 ± 0.94). Similar to the linear analysis, the techniques of DT and MT did not present statistically significant difference between each other for the angular deviation between implants planned and installed ( Fig. 5B ).

## Discussion

This in vitro study aimed to compare the effectiveness of the three following surgical techniques for installing osseointegrated implants: DT, MT, and conventional installation of implants with nonprototyped surgical guide. After comparing the angular and linear deviations obtained in the implants installed with the three techniques, it was observed that the conventional implant installation with the nonprototyped surgical guide presented greater discrepancy between the final positioning and the one planned in this study. Moreover, the DT and the MT techniques presented similar performance regarding the angular and linear deviations analyzed. Both techniques assessed in this study using CT, and the prototyped guide presented smaller discrepancies in the final implant positioning relative to the planned one, in comparison with the conventional technique.

Previous in vivo studies comparing the clinical and radiographic results of dental implants installed with the conventional technique and guided surgery showed that both techniques presented satisfactory performances. 27 28 29 30 Additionally, implant installation with a detailed planning and the use of prototyped guides ensured the predictability of results for the implants installed using the flapless technique. 27 31 32

Therefore, the higher precision on implant placement using the guided surgery technique observed in this study, and its satisfactory clinical performance suggests that guided surgery presents advantages over the conventional one because it allows the following: (1) reducing errors associated with conventional freehand implant placement; (2) reducing the risk of affecting critical anatomical structures; (3) performing a less invasive and flapless surgical approach, reducing postoperative complications and discomforts; and (4) integrating prosthetic planning and the implant installation procedure. 13 29 33 34 35 36 37 38 39 40 41 42 However, the guided surgery technique involves a complex planning susceptible to errors that may result in deviations between the positioning planned and the postoperative location of the implant. 15 33 43

The accuracy in implant positioning using guided surgery was assessed in a meta-analysis that showed a mean horizontal deviation of 1.1 to 1.6 mm and angulation of 5.26 degrees observed in clinical, in vitro, and cadaver studies. 39 Vercruyssen et al (2014) 30 assessed the accuracy of guided surgery compared with mental navigation or the use of a pilot-drill template in fully edentulous patients through vertical (depth) and horizontal (lateral) deviations. Their results showed that the nonguided surgery has inaccuracy which is significantly higher. 30 Aly et al 43 assessed interarch and linear deviations, comparing the accuracy of 3D printed casts, their digital replicas, and conventional stone casts. Their results showed that the digital casts produced significantly higher error than the other two groups in all linear and interarch measurements, and the 3D printed casts has clinically acceptable accuracy. 43

Vermeulen 38 assessed angular and linear deviations, comparing the conventional and guided techniques in the anterior maxillary region, concluding that guided surgery presents more precision and predictability. In the same research, 38 the author also compared the precision of guided surgery and free hand in the installation of single and multiple implants, and concluded that with guided surgery, there are less angular and linear deviations, but in relation to vertical deviations, the results were similar.

In contrast, Behneke et al investigated the factors affecting the accuracy of guided surgery, comparing angular and linear deviations between the positioning planned for implants and the one assessed after surgery. 33 The deviations observed were similar for implants positioned in the maxilla and mandible, as well as with flapless approaches. However, larger deviations were observed in multiple edentulous spaces in comparison to isolated missing teeth. Moreover, using the prototyped guide ensured higher accuracy in the implementation of the virtual planning when comparing with the conventional technique. A cadaver study affirmed that the safe margin of error of linear deviation is a maximum of 1 mm. 40

In this in vitro study, angular and linear deviations were observed in both techniques using the prototyped guide, but they were smaller than in the conventional technique for implant installation. However, the report on minimum deviations in the positioning planned for implants using guided surgery were not associated with the causes of treatment failure.

Vercruyssen 30 and Lopez et al 42 report in their studies that the biggest challenge of guided surgery is deviations in the apical level (depth). In this way, precautions should be taken regarding the maximum deviations in the apical level of implants installed close to critical anatomical structures. Clinical factors such as the presence of artifacts in the CT, the length of the implant planned, and the stabilization of the guide during the surgical procedure should be assessed when planning and executing the surgical technique. 27

Despite the advantages discussed until now, the guided surgery results in higher temperatures in the bone tissue than the conventional technique. 44 This temperature increase is associated with the absence of direct irrigation, which is only performed outside the prototyped guide. However, a study in rabbits showed that heating was associated directly with the number of times the perforation drill of the bone tissue was used, and the regular exchange of drills is required to prevent overheating the bone tissue. Moreover, despite more heating, the guided surgery did not produce sufficient heat to cause bone necrosis. 44

This in vitro study did not show differences in angular and linear deviations between the dual scanning and the MT techniques. The degree of deviations found does not interfere with prosthesis production. Both techniques presented similar and superior performance to the conventional technique. It is worth noting that there were neither in vitro nor in vivo studies using the MT technique, which complicated the discussion of the data obtained in this study. However, regarding the number of clinical steps, number of tomographies performed, cost, and practicality, the MT technique is suggested to present advantages over the dual scanning technique. Further in vivo studies should be performed to confirm the findings of the present study, considering the mannequin has a homogeneous bone density and a standardized topography.

## Conclusion

This *in vitro* study concludes that the techniques of dual scanning and MT presented smaller angular and linear deviations than the conventional technique with the nonprototyped guide. Moreover, there was no difference between both guided surgery techniques, but the MT technique presents clinical advantages with lower cost and higher practicality and speed.
